# Effects of Physical Activity on Children's Motor Skill Development: A Systematic Review of Randomized Controlled Trials

**DOI:** 10.1155/2020/8160756

**Published:** 2020-12-30

**Authors:** Daniel J. McDonough, Wenxi Liu, Zan Gao

**Affiliations:** School of Kinesiology, University of Minnesota-Twin Cities, Minneapolis 55455, USA

## Abstract

**Objective:**

This systematic review synthesized current randomized controlled trials (RCTs) examining casual evidence regarding the effects of traditional and exergaming-based physical activity (PA) interventions on motor skill development in typically developed children (i.e., those aged 6-12 years).

**Methods:**

We adhered to the PRISMA-P statement and searched electronic databases (Medline, PsycInfo, Web of Science, PubMed, ERIC, Scopus, and SportDiscus) from inception through July 2020. We screened for peer reviewed RCTs published in English between 2000 and 2020 examining the effect of PA on motor skill development in healthy children.

**Results:**

A total of 25 RCTs were included, 20 (80%) of which reported significant improvements in children's motor skill performance. Specifically, 18 studies examined traditional PA interventions and 7 studies examined exergaming-based PA interventions, 83% and 71% of which observed statistically significant improvements in children's motor skill development, respectively.

**Conclusions:**

Findings support the causal evidence regarding the effects of PA on motor skill development in children. Notable limitations of this review included heterogeneity of measurement protocols and assessment tools used to test children's motor skills across studies, a wide range of PA intervention dose across studies, and the lack of power analyses and long-term follow-up assessments in individual studies to discern appropriate sample sizes and long-term effectiveness, respectively. To further strengthen the evidence in this emerging field, we advocate for future RCTs to employ a priori power analyses, long-term follow-up measurements, and more exergaming-based interventions to allow for comparisons with traditional PA interventions, to explore the dose response and moderating relationships between PA and motor skill development in childhood, and to utilize homogenous assessment instruments to allow for more rigorous, quantitative syntheses.

## 1. Introduction

Currently in the U.S., approximately 33% of children and adolescents have opverweight or obesity [1]. The overweight and obesity epidemic has become a major public health challenge given weight-associated chronic diseases account for 70% of deaths and 85% of health care costs, annually [2, 3]. Physical inactivity is among the top contributors to this issue [4] as only about 25% of U.S. youth meet recommended physical activity (PA) levels [5, 6]. Fortunately, unlike other major contributing risk factors (e.g., pollution and medications), physical inactivity is a modifiable behavioral risk factor meaning this behavior can be changed [4]. Therefore, since PA and health behaviors are learned in the developmental years and track into adulthood [7], it is vital to establish lifelong, healthy PA habits during childhood. Because learning how to properly move is a necessary skill underlying PA behaviors in children [8, 9], interventions targeting the development of motor skills often precede those which target general PA promotion. However, given that less than 25% of children meet recommended PA levels [5, 6] and given the reciprocal determinism between PA and motor skills [8, 9], interventions have more recently focused on general PA promotion among these populations with the aim of increasing motor skills and ultimately increasing long-term PA adherence and health.

Motor skills have been operationally defined as sequences of learned movements that when combined yield smooth and efficient movements which leads to specific task mastery [10]. More broadly, fundamental motor skills include both fine and gross motor skills, the latter of which encompasses locomotor skills (e.g., running, hopping, and jumping), object control skills (e.g., kicking and throwing), and body coordination (e.g., balance control) [11]. Notably, however, these categories are not exclusive, and thus, motor skills from one category may take place concurrently with elements of other categories [12]. Cross-sectional and longitudinal evidence has supported the reciprocal and dynamic relationship between PA and motor skills [13–18], and compiling evidence has indicated the development of motor skills to improve various health indices in children including cardiorespiratory fitness, muscular strength and endurance, and perceived competence, to list a few [19, 20]. As such, the development and employment of PA interventions targeting improved motor skills in children have become an emerging field of inquiry [21].

Accordingly, as more PA interventions examining this relationship accumulate, more recent and thorough reviews are needed to discern the overall effectiveness of PA interventions on children's motor skill development. One such review was conducted in 2009 but included research designs other than randomized controlled trials (RCTs) and, therefore, was unable to infer causal relationships. Additionally, recent public health efforts have been aimed at integrating modern technologies into PA interventions to gauge children's interest [22], and given the rapid evolution of technology in the past decade, the review did not include technology-based interventions. Given its requirement for gross motor activity to participate [21], exergaming is one technology-based intervention strategy which has shown promise in the promotion of motor skill development in child rehabilitation settings [23] and in nontypically developing children [24]. Another recent review was conducted examining the effect of PA interventions on motor skill development in early childhood (i.e., those aged 3-5 years), thereby missing the opportunity to examine the effects PA interventions in childhood (i.e., those aged 6-12 years)—a critical developmental period and the last chance to establish PA behaviors before reaching adolescence where nonschool and leisure-time PA levels tend to significantly decline [25, 26].

Based on the preceding literature review, we developed the following research question: based on RCT-based evidence, are traditional and exergaming-based PA interventions effective for improving motor skill development in typically developed children? Therefore, the purpose of our study was to address these gaps in the literature and to systematically evaluate the current RCT-based evidence examining the effects of traditional and exergaming-based PA interventions on healthy children's motor skill development. Findings from this review will help to better inform scholars, physical educators, and other health professionals of the benefits of regular PA participation on children's motor skills and strengthen the development of empirically based PA guidelines for this age group.

## 2. Materials and Methods

We followed the Preferred Reporting Items for Systematic Review and Meta-Analysis Protocols (PRISMA-P) statement for reporting of this review [27].

### 2.1. Information Sources and Search Strategies

We searched the following electronic databases for relevant literature: Medline, PsycInfo, Web of Science, PubMed, Education Resources Information Center (ERIC), Scopus, and SportDiscus, as well as Google Scholar. All investigators (D.M., W.L., and Z.G.) collaborated and searched literature by applying the following search terms in all possible combinations: (“physical activity” OR “exercise” OR “sports program” OR” “physical education” OR “exergaming” OR “active video game”) AND (“motor skill” OR “motor skill competency” OR “motor coordination” OR “motor development” OR “motor function” OR “motor performance” OR “motor abilities” OR “fine motor skills” OR “gross motor skills” OR “locomotor skills” OR “object control skills”).

### 2.2. Eligibility Criteria

We applied the following inclusion criteria with reference to the participants, interventions, comparisons, outcomes, and study design (PICOS) guidelines: (1) study sample consisted of healthy, normal developing children (6-12 years) without motor or mental impairments (e.g., motor disabilities, autism spectrum disorders); (2) the study assessed the effects of a traditional or technology-based PA intervention against a control group; (3) the study employed quantitative fundamental motor skills assessments; and (4) the study employed a RCT. Moreover, we only included empirical, peer-reviewed research published in English between January 2000 and October 2020 and other study designs (e.g., cohort and cross-sectional) were retrieved but excluded from the analysis.

### 2.3. Data Extraction

Three investigators (D.M., W.L., and Z.G.) independently screened all potential articles by evaluating the titles, and if able to discern study relevance, we evaluated the abstracts. Data extraction was completed by one investigator (W.L.) and checked for accuracy by another (D.M.). We then created list of relevant published articles in a Microsoft Excel spreadsheet. In detail, we extracted the following information: (1) publication year and the country the research was conducted in; (2) details of study methodology (i.e., study design, sample characteristics, study duration, type of PA intervention employed, study outcomes, and instruments used); and (3) key findings regarding the effectiveness (or lack thereof) and potential of PA on children's motor skills. Finally, we cross referenced the bibliographies of selected articles to further identify relevant studies. Noteworthy is the fact that we were not blinded to the authors or journals of the included articles and we made no attempts to contact study authors or correspondents to acquire missing information.

### 2.4. Risk of Bias within and across Studies

Two investigators (D.M. and W.L.) independently assessed the risk of bias within each included study. Specifically, we rated each study using an 8-item quality assessment tool used in previous literature reviews of predominantly field-based RCTs [28, 29]. Notably, we used this tool because other tools for assessing bias in RCTs (e.g., Cochrane Risk of Bias 2.0) contain domains which are more applicable to clinical trials (e.g., allocation concealment, blinding of participants, and personnel) whereas this tool uses domains like employment of validity measures and follow-up assessments which we deemed more appropriate for field-based PA interventions. We rated each within-study item as “positive” if the item was present and explicitly described or “negative” if the item was absent or inadequately described. To ensure reliable scoring, two investigators (D.M. and W.L.) independently scored the risk of bias of each study within the quality assessment. If necessary, disagreements were adjudicated by a third reviewer (Z.G.). We calculated final quality scores for each study by summing all “positive” scores. Studies were considered high-quality when they scored above the median score (i.e., 7) following the scoring of all included studies. For the risk of bias across studies, the domains which we agreed may affect the cumulative evidence most were the employment of validity measures and participant retention given the variety of tools available for assessing children's motor skill development and the need for the ability to maintain children's interest in the employed PA interventions to promote long-term PA behaviors and motor skill development, respectively.

## 3. Results

### 3.1. Study Selection

**Figure 1:**
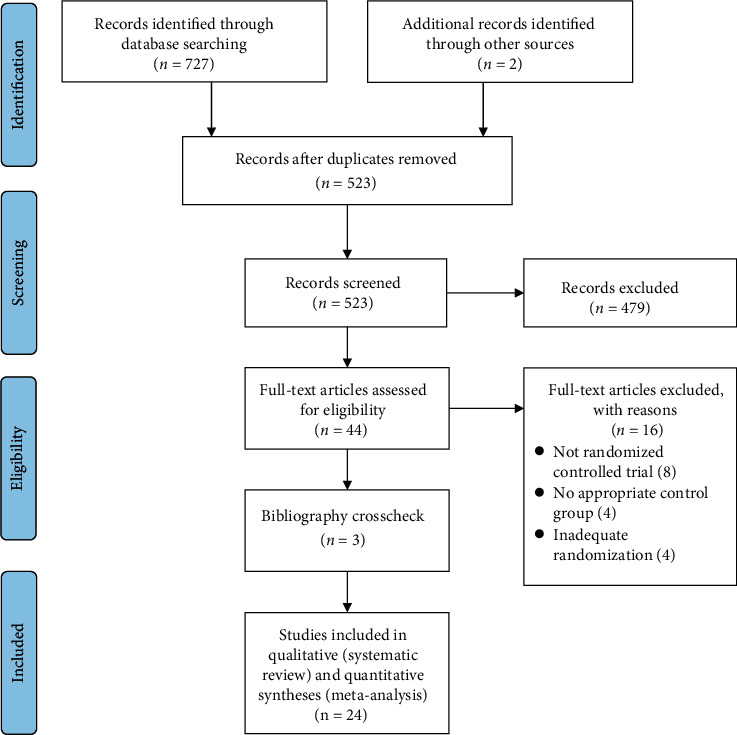
PRISMA flow diagram of studies through the review process.

Through a search of the databases, we identified a total of 727 potential articles. Following the removal duplicate articles, two investigators (D.M. and W.L.) screened the titles and abstracts of the remaining articles to further identify potentially relevant articles. An additional 3 studies were located through bibliography crosschecks. After thorough assessment of all full-text articles, 25 studies met all of the *a priori* established inclusion criteria and were included in this systematic review (see Figure 1). Reasons for excluding potential articles included ineligible age (i.e., those < 6 and >12 years), special populations (e.g., those with chronic disease), did not include measures of motor skills, and non-English language articles. Noteworthy is that we observed high interrater agreement such that 24 of 25 (96%) of the included articles were agreed upon and obtained between the study investigators.

### 3.2. Study Characteristics

Characteristics of all included studies are shown in Table 1. Of the 25 RCTs, 7 (28%) studies examined the effects of exergaming-based physical activity interventions on children's motor skills [30–36] and the remaining 18 (72%) assessed the impact of traditional PA interventions on children's motor skills [33, 37–54]. The studies were conducted in different countries: 6 in Australia [30, 33, 45, 46, 50, 53], 3 in the United States [31, 38, 39], 3 in China [37, 44, 51], 3 in the United Kingdom [40, 43, 47], 2 in Canada [35, 36], 2 in Greece [32, 48], 2 in the Netherlands [34, 54], 1 in Ireland [42], 1 in Switzerland [41], 1 in Italy [52], and 1 in Norway [49]. Among these studies, 20 were conducted in the school setting [30–39, 42–49, 53, 54], 1 was conducted in a home-based setting [40], 1 was conducted in a laboratory setting [51], 1 was conducted in a childcare center [41], 1 was conducted in a field-based (sports) setting [52], and 1 was conducted in a community-based setting [50]. Notably, most of the studies were published after 2010, except for 1 study that was published in 2002 [48] and 2 studies that were published in 2008 [43, 53], and 16 (64%) of the studies were published after 2015 [30–33, 37–40, 42, 44–46, 49, 51, 54], indicating that high-quality research examining PA interventions on children's motor skill development is an emerging scientific field of inquiry.

Further, we observed a relatively large variability in sample size (*n* = 34 to 891) and intervention length (4 weeks to 12 months) across studies. The exposure in most (72%) of the studies was a traditional PA/exercise program or class followed by exergaming-based PA interventions while the control conditions were most often usual care or regular school curriculum (i.e., no PA intervention). Although motor skill development measurement tools varied across studies, they were most often direct observations made by trained research assistants or assessments directly completed by the children. Gross motor skills, locomotor, and object control skills were the most commonly assessed outcomes in the assessment of motor skill performance. In this review, we did not employ a meta-analysis due to the heterogeneity of both exposures and outcomes across the included studies.

### 3.3. Study Quality and Risk of Bias Assessment

Scores of study quality/risk of bias for all individual studies ranged from 6 to 8 with a median score of 7 (Table 2). An individual study was considered high quality/low risk of bias when it scored above the median score of 7, moderate quality/medium risk of bias if scored at the median score of 7, and low quality/high risk of bias if scored below the median score of 7. In detail, 5 studies (20%) received an overall rating of strong quality/low risk of bias, 7 studies (28%) received an overall rating of moderate quality/medium risk of bias, and 13 studies (52%) received an overall rating of weak quality/high risk of bias. Noteworthy is the fact that all studies succeeded in retaining at least 78% of the participants. The most common issues with the study quality/risk of bias were lack of power calculations for appropriate sample sizes and a lack of follow-up measurements, respectively. Regarding bias across studies for the 2 primary domains, 25 studies (100%) sufficiently reported on intervention fidelity and retention and as previously mentioned; all studies had high participant retention rates (≥78%). Further, 25 studies (100%) employed valid measures of assessing children's motor skill development, the majority of which used the Test of Gross Motor Development-Second Edition (TGMD-2).

### 3.4. Measurement Protocols

Various types of instruments were used to measure motor skills. Specifically, the most commonly used instrument in assessing children's motor skills was the TGMD-2, followed by the TGMD-Third Edition and the original TGMD, the Victorian Fundamental Motor Skills Assessment Instrument, the Körperkoordinations für Kinder (KTK) test, the Bruininks-Oseretsky Test of Motor Proficiency, Second Edition (BOT-2), the Fundamental Motor Skills Quotient (FMSQ), and the Zurich Neuromotor Assessment (ZNA). Notably, measurement tools used for motor skills varied across studies. Typically, assessments were directly completed by children or through direct observations made by trained research assistants. Although different instruments were used across various studies, validities of these assessments have been proven when being applied to children within the school setting (Table 1).

### 3.5. The Effectiveness of PA on Motor Skill Development

Overall, of the 25 RCTs examining the effects of PA interventions on children's motor skill development, 20 (80%) reported statistically significant improvements from pre- to postintervention [31, 32, 34–39, 42–53]. More specifically, of the 7 studies examining the effects of exergaming-based PA interventions on children's motor skill development, 5 (71%) observed significant intervention effects and of the remaining 18 studies which employed traditional PA interventions, and 15 (83%) observed significant intervention effects on children's motor skill development. Notably, of the 5 studies which reported no statistically significant changes in children's motor skill development, 2 were long-term interventions (9-12 months) conducted outside of the school setting (1 home-based intervention [40] and 1 childcare center intervention [41]) and the other 3 were short-term interventions [30, 33, 54] (6-14 weeks) performed in the school setting. However, of the 5 studies showing no effects of PA on children's motor skill development, no study reported detrimental effects of increased PA on motor skill development. That is, PA interventions did not adversely affect children's motor skill development.

## 4. Discussion

The purpose of this review was to synthesize and comprehensively evaluate all published RCTs examining the causal relationship between traditional and exergaming-based PA interventions on the motor skill development of healthy children aged 6-12 years. Twenty-five studies met the inclusion criteria and were included in the final analysis. Overall, findings suggested that increased PA had significant positive effects on children's motor skill development. More studies examined traditional PA interventions compared to exergaming-based PA interventions, but both showed relatively high effectiveness on children's motor skill development. Lastly, no study observed increased PA duration or frequency to have a detrimental effect on the development of children's motor skills.

Overall, the majority (80%) of studies observed beneficial effects of PA promotion interventions on children's motor skill development, the majority of which were conducted within a school setting. Of the 5 studies which reported no significant effects, 40% were conducted outside of the school setting (1 home-based intervention [40] and 1 childcare center intervention [41]). Thus, we postulate that PA promotion interventions are more effective at increasing children's motor skill development when conducted in the school setting. Indeed, given the amount of time children spend at school and the structured schedules within these settings, it is well-documented that schools have the greatest influence on children's PA behaviors [55] and school-based PA has been observed as a strong predictor of children's total daily and weekly PA [56] and is positively associated with higher levels of daily moderate-to-vigorous intensity PA [57]. That said, we are not surprised that motor skill development was not significantly enhanced in childcare or home-based settings given there is less PA-related structure and study adherence (PA participation in this case) is less controlled [58]. Indeed, one study reported dropout of intervention participants [40], and the other noted the complexity of conducting PA interventions outside of a study setting and how intervention fidelity suffered as a result [41]. Additionally, these two interventions were long-term (9-12 months) and without structure, and participants likely lost interest in the intervention within the timeframe and PA participation (and motor skill development) likely suffered because of this. Notably, 2 studies which were conducted outside of a school setting observed significant results (1 laboratory-based [51] and 1 sports setting [52])—settings which also have more structure and control of participants' adherence and fidelity outcomes. Indeed, previous research has demonstrated and noted the importance of structure when aiming at promoting children's motor skill development [59–62].

Another possible mediating factor in the relationship between PA and motor skill development in children is PA dose (i.e., the frequency and amount of time devoted to the instruction and practice of motor skills [63]). Indeed, there was distinct homogeneity in intervention length across the included studies which ranged from 4 weeks to 12 months. Specifically, of the 5 studies which did not observe significant intervention effects on children's motor skill development, heterogeneity was also present as intervention length ranged from 6 weeks to 12 months, making it difficult to assess whether PA dose was responsible for the mixed findings. Taken together, previous research has demonstrated inconsistencies with regard to PA dose on children's motor skill proficiency. For example, similar to the findings of this review, individual studies have demonstrated significant effects on children's motor skills after a 540-minute PA dose [59–61] whereas other studies observed statistically nonsignificant findings after 400- and 3600-minute PA doses [53, 64]. One study [63] directly examined the dose-response relationship between PA dose using the same intervention and young children's motor skill development (tested using the TGMD-2) and divided the participants into 1 of 4 groups based on PA dose: (1) 660 minutes, (2) 720 minutes, (3) 900 minutes, and (4) control. Interestingly, the researchers found that all 3 dosages resulted in significantly greater improvements in motor skill performance compared to control, with no significant differences between PA dosages. However, this study was conducted in preschool-aged children and it remains unclear how PA dose affects motor skill development in children.

To the best of our knowledge, this is the first systematic review to examine the causal relationship of traditional and exergaming-based PA RCT interventions on children's motor skill development. We applied strict inclusion criteria and only included high-quality RCTs among a homogenous sample of healthy children. Additionally, we identified participant retention as a potential major risk of bias across studies and 100% of the included studies sufficiently discussed intervention fidelity and participant retention and all studies were able to retain ≥78% of participants, thereby strengthening the cumulative evidence of this review. However, this review is not without limitations, and accordingly, educators and other health practitioners should interpret the results with caution. First, for logistical reasons, we only included peer-reviewed studies published in English when non-English publications, and therefore further comparative evidence, may have been available on the topic. However, language restriction does not consistently bias the results of narrative or quantitative syntheses [65]. Second, there was noticeable heterogeneity of measurement protocols and assessment tools used to test children's motor skills across studies. Nevertheless, validated testing instruments were used across all studies which minimized a major domain of bias and further strengthened the overall evidence of this review. Third, likewise, there was some heterogeneity in the dose of PA administered across studies such that some were acute interventions and some were long-term interventions and some only intervened 1 day per week whereas others intervened 5 days per week. In addition, individual studies and the review as a whole did not assess possible moderating effects of PA on children's motor skill development. For example, study setting (e.g., home- vs. school-based) or PA intervention type (e.g., exergaming vs. traditional PA) may have moderated the effectiveness of the PA interventions on children's motor skill development. Lastly, 2 major sources of within-study risk of bias were lack of a power analysis and lack of long-term follow-up testing. In detail, we observed over half of the included studies to be of low quality/high risk of bias due to a lack of power analysis to determine appropriate sample sizes and a lack of follow-up observations to track the long-term effectiveness of the employed interventions. Thus, we suggest future RCTs in this field of inquiry to address these gaps in study design to strengthen the quality of available evidence and to better establish the long-term effectiveness of PA promotion interventions on children's motor skill development.

## 5. Conclusions

Overall, findings suggested a causal relationship between increased PA and improved motor skill development in children, especially when interventions were conducted in a school setting [66]. Although traditional PA intervention strategies have been examined in the literature more than exergaming-based PA intervention strategies, exergaming interventions showed similar effectiveness relative to the number of available trials, and given their enjoyable and innovative nature and ability to leverage children's interest in videogame play, they hold promise for future motor development in this population. These findings have important public health implications as they help to inform educators and other health practitioners that regardless of the employed PA promotion strategy (traditional or exergaming-based PA), simply getting older children to move more can enhance their motor skill development which, in turn, may help establish healthy PA behaviors which track into adulthood [7] and, ultimately, help attenuate the grand challenge of adult overweight and obesity in the U.S. [67].

Therefore, if school funding allows, we recommend teachers and/or physical educators implement exergaming stations within the school to allow students to engage in enjoyable PA before, during, and/or after school to increase PA output and, ultimately, motor sill development. If school funding is not available, other free and creative PA promotion strategies should be used, such as intermittent learning breaks wherein short bouts of PA are integrated throughout the day during class. Likewise, parents should consider implementing exergames in their homes to help encourage PA participation *outside* of the school setting and/or encourage their children to play traditional games that require PA. Nevertheless, our study shows that whether in the school setting or outside of the school setting and whether traditional or technology-based PA, simply getting children to move more and engage in PA is beneficial to improving motor skill development which may lead to greater PA-related self-confidence and healthy PA behaviors that track throughout the lifespan.

## Figures and Tables

**Table 1:** Characteristics of individual studies included in the review.

Study	Sample	Design/setting	Outcome/instrument	Exposure	Dose	Findings
Barnett et al. [30]; Australia	Children aged 4-8 years; *n* = 95	RCT School	Object control skills; perceived object control skills	Intervention children played exergaming (Wii), while control children have no treatment	1 hour per week for 6 weeks	Object control improved overtime, but there was no significant group difference; notably, intervention children found exergaming sessions were more enjoyable
McGann et al. [31]; US	Children aged 5-7 years; *n* = 40	RCT School	Locomotor skills Measured by TGMD-2	Intervention children participated in a purpose-built exergame, while control children participated a commercial exergame	3-minute high intensity gameplay each day, 5 days a week, for 8 weeks	Intervention children showed significant improvement for each locomotor skill (run, hop, skip, and slide), while the control group intervention had significant improvement in only one locomotor skill (the slide)
Chan et al. [37]; China	Primary school children, mean age 8.4 years; *n* = 282	Cluster RCT School	FMS competence, perceptions of physical and movement skill competence, teacher support, and enjoyment FMS was measured by TGMD-3 The athletic competence subscale of the Self-Perception Profile for Children (SPPC–6 items) was used to assess children' subjective evaluation of their athletic ability	Intervention children received an assessment-based teacher-led FMS intervention, while control children remain in their usual PE lessons	A total of 550 minutes, for a period of 13 weeks	Significant intervention effects were found for locomotor skills and perceived teacher support. However, there was a group-by-time effect for perceived physical competence in favour of the control group
Lee et al. [38]; US	Children aged 5-8 years; *n* = 36	RCT School	FMS competence and MVPA FMS was measured by TGMD-2 MVPA was measured by accelerometers	Intervention children participated in FMS-based need supportive afterschool program, while control children participated in a regular unsupervised afterschool program	60 min per session, 3 times per week, for 8 weeks	Intervention children had significant improvement in FMS competence and MVPA compared to control children; there was no gender difference regarding the FMS competence and MVPA
Cohen et al. [39]; US	Primary school children aged 7-10 years; *n* = 460	Cluster RCT School	MVPA, cardiovascular fitness, FMS competence, perceived sport competence MVPA was measured by accelerometer FMS competence was measured by TGMD-2 Perceived sport competence was assessed using the perceived competence sub-scale from Harter's Self-Perception Profile	Intervention children participated in a multicomponent PA and FMS intervention, while control children remained in their usual PE and school program	12 months	Changes in MVPA were associated with changes in object control skills, overall FMSs, and perceived competence Overall FMSs had a significant mediating effect on MVPA. In addition, overall FMSs and locomotor skills had a significant mediating effect on cardiovascular fitness
Laukkanen et al. [40]; UK	Children aged 4-7 years; *n* = 91	RCT Home-based	PA and motor competence PA was measured by accelerometer Motor competence was measured by KTK (Körperkoordinations für Kinder) and throwing and catching a ball (TCB) protocols	Parents in the intervention group families received a tailored counseling to increase children's PA, while control group families did not receive any counseling	One-year intervention	The results showed significant decrease of MVPA in the intervention group when compared to the control group (*p* < 0.05). The TCB showed a nearly significant improvement at six months in the intervention group compared to the controls (*p* = 0.051), but not at 12 months. The intervention group had a steadier development of the KTK when the interaction of season was taken into account
Bonvin et al. [41]; Switzerland	Childcare center children with mean age 3.3 years; *n* = 648	Cluster RCT Childcare center	Motor skills BMI, PA, and quality of life Motor skill measures were adapted from the Zurich Neuromotor Assessment (ZNA) test PA was measured by accelerometer Quality of life of the participating children was assessed using the parent report for children of PedsQL 4.0	The intervention included training of the educators, adaptation of the childcare built environment, parental involvement, and daily physical activity, while the control group had no treatment	9 months	There was no significant improvement in motor skills compared to the control group; notably, not all childcare centers implemented all the intervention components
Vernadakis et al. [32]; Greece	Elementary school children aged 6-7 years; *n* = 66	RCT School	Object control skills The object control skills were measured by TGMD-2	Two intervention groups: (1) exergame-based training program and (2) traditional training program The control group did not receive any treatment	8 weeks; 2 times per week; 30 minutes per session	Significant improvement in object control skills in both intervention groups, but not in the control group. There was no significant difference regarding the improvements between two intervention groups
Costello and Warne [42]; Ireland	3^rd^ and 4^th^ grade school children aged 8-9 years; *n* = 100	RCT School	Motor skills Measured by fundamental motor skill quotient	Intervention children received FMS lessons, while control group children did not receive any treatment	4 weeks; 2 times per week; 30 minutes per session	Intervention children had significant improvements in FMSs, but not in control group
Johnson et al. [33]; Australia	Children aged 6-10 years; *n* = 36	RCT School	Object control skills and competence Object control skills were measured by TGMD-3; perceived object control skill was measured by the Pictorial Scale of Perceived Competence for Young Children	Intervention children received weekly exergaming intervention, while control group children did not receive any treatment	6 weeks; 50 min per week	No significant differences between the control and intervention groups were observed for both outcomes
Foweather et al. [43]; UK	Children aged 8-9 years; *n* = 34	RCT School	FMS Skills were measured by process-oriented measures with video analysis	Intervention children participated in an after-school FMS training program, while control group children remain normal routine	9 weeks; one hour per session; 2 times per week	Significant improvement in static balance, but no significant improvements in other skills
Sit et al. [44]; China	Children aged 6-10 years; *n* = 131	RCT School	FMS proficiency, PA, self-perceived competence FMS was measured by TGMD-2, PA was measured by accelerometer (GT3X+), and self-perceived competence was measured by Physical Self-Descriptive Questionnaire (PSDQ)	The experimental group received a training program focusing on the practice of five specific FMS skills (running, jumping, catching, kicking, and throwing) Control group children received regular PE lessons	8 weeks; 40 minutes per week	FMS training resulted in significantly improved FMS proficiency and increased PA and enjoyment of activity participation in children
Lander et al. [45]; Australia	Year 7 girls aged 11-13 years; *n* = 190	Cluster RCT School	FMS Skills were measured by Victorian FMS assessment instrument	Intervention children received teacher-led FMS-focused training, while control group children remained normal PE	12 weeks; 90 minutes per week	Significant improvements in object control skills and total skill compared to the control group
Lander et al. [46]; Australia	Year 7 girls aged 11-13 years; *n* = 200	Cluster RCT School	FMS Skills were measured by Victorian FMS assessment instrument	Intervention children received teacher-led FMS-focused training; while control group children remained normal PE	12 weeks; 90 minutes per week	Significant improvements in perceived motor skill competence and actual motor skills
Johnstone et al. [47]; UK	Elementary school children; *n* = 137	Cluster RCT School	FMS, PA, inhibition, and math fluency FMS was measured by TGMD-2, PA was measured by accelerometer, inhibition was measured by Flanker Test, and math fluency was measured by One Minute Basic Facts Test	Intervention children received outdoor PA session, while control group had no treatment	10 weeks; one hour per week	Active play sessions were shorter than planned on average by 10 min, and participants spent a mean of 39.4% (14.2) of the session time in MVPA. There was preliminary evidence of a small intervention effect on MVPA (*d* = 0.3), FMS score, inhibition, and math fluency
Karabourniotis et al. [48]; Greece	Children aged 5-7 years; *n* = 45	RCT School	FMS, content areas of PE courses FMS was measured by TGMD, PE content was measured by the Academic Learning Time-Physical Education	Intervention children received a skill-oriented program, while control children received regular school PE	12 weeks	Intervention children had significant FMS improvements compared to the control group
Mombarg et al. [34]; Netherlands	Children aged 7-12 years; *n* = 29	RCT School	Balance skills Measured by M-ABC-2 and the Bruininks-Oseretsky test of motor proficiency (BOT-2)	Intervention children received Wii exergaming, while control children did not receive any treatment	6 weeks; 30 minutes per session; 3 times per week	Intervention children had significant improvements in balance skills compared to the control group
Aadland et al. [49]; Norway	Children aged 10 years; *n* = 123	Cluster RCT School	Executive function FMS was measured by TGMD-2	The intervention constituted three PA elements: PA educational lessons, PA breaks, and PA homework, adding 165 minutes of PA to the mandatory 135 minutes of PA and physical education	7 months	There was no effect of the intervention on executive functions in the intention-to-treat analyses. Per protocol analyses revealed small effects of the intervention on the composite score of executive functions, cognitive flexibility, and motor skills
Cliff et al. [50]; Australia	Children aged 5-9 years; *n* = 132	RCT with 6- and 12-month follow-up Community-based	FMS, perceived athletic competence, PA, screen behaviors FMS was measured by TGMD-2, perceived competence was measured by Self-Perception Profile for Children, PA was measured by accelerometer, and screen behaviors were measured by the Children's Leisure Activities Study Survey	Children were randomly assigned to three groups: (1) a child-centered physical activity skill development program (Activity), (2) a parent-centered dietary modification program (Diet), and (3) a combination of both programs (Activity+Diet)	6 months; 10 2 h weekly group sessions (~90 min of physical activity per session) and weekly “home challenge” activities	The findings indicated that the PA and PA+Diet groups had significant improvements in FMS
Pan et al. [51]; China	Children aged 7-12 years; *n* = 60	RCT Clinic	FMS, executive function FMS was measured by TGMD-2, and executive function was measured by Stroop Color and Word Test and Wisconsin Card Sorting Test	Intervention children received table tennis and physical and cognitive training, while control group did not receive treatment	12 weeks; 70 minutes per week	Training group had significant improvements in both locomotor and object control skills compared to the control group
Piazza et al. [52]; Italy	Female rhythmic gymnasts, aged between 10 to 13 years, *n* = 57	RCT Field-based	Squat jump test, counter movement jump test, hopping test, flexibility of the hip	Participants were randomly assigned to the unspecific resistance training group or specific resistance training group	6 weeks 2 days per week (nonconsecutive days)	The main result was that both unspecific resistance training and specific resistance training protocols positively affected the jumping performance, with an increase of the lower limb explosive strength of 6-7%, with no side effects No significant differences were detected among groups for flexibility, body mass, calf, and thigh circumferences
Salmon et al. [53]; Australia	School children aged 10-11 years; *n* = 311	RCT School	BMI, PA, screen behaviors, PA enjoyment, FMS PA was measured by accelerometers, screening behaviors were assessed by the self-reported screen behaviors questionnaire, and FMS was measured by TGMD	Children were randomized by class to one of the four conditions: a behavioral modification group (BM); a fundamental movement skill group (FMS); a combined BM/FMS group (BM/FMS); and a control (usual curriculum) group	One school year Each of the intervention conditions consisted of 19 lessons (40–50 minutes each)	There was a significant intervention effect from baseline to post intervention on age- and sex-adjusted BMI in the BM/FMS group compared with controls, which was maintained at 6- and 12-month follow-up periods. Compared with controls, FMS group children recorded higher levels and greater enjoyment of PA
Sheehan and Katz [36]; Canada	Children aged 6-10 years; *n* = 67	RCT School	Balance	Intervention children participated in a Wii Fit exergaming program, while control group children participated in traditional PA	6 weeks; 3 days per week	The intervention children had significant improvements in postural stability compared to the control group
Sheehan and Katz [35]; Canada	Fourth grade children; *n* = 64	RCT School	Balance	Intervention children participated in a iDance exergaming program, two control groups were used: (1) a physical education (PE) class geared toward agility, balance, and coordination (ABC) improvement and (2) a typical PE curriculum class	6 weeks; 34 min per day; 4-5 days per week	Exergaming students improved their postural stability significantly over a 6-week period compared to those in the typical PE class
van der Fels et al. [54]; Netherlands	Children aged 7-10 years (grades 3-4); *n* = 891	Cluster RCT School	Gross motor skills were assessed using the Körperkoordinations für Kinder test Bruininks-Oseretsky Test of Motor Proficiency, Second Edition (BOT-2), was used to include a measure for ball skills	Intervention children participated	Intervention groups received aerobic or cognitively engaging exercise (14 weeks, four lessons per week). Control groups followed their regular physical education program	No main effects of the aerobic intervention and the cognitively engaging intervention on cardiorespiratory fitness and motor skills in primary school children in grades three and four

**Table 2:** Risk of bias/quality of individual studies included in the review.

Articles	Randomization	Control	Pre-post	Retention	Missing data	Power analysis	Validity measure	Follow-up	Score	Effectiveness
Barnett et al. [30]	+	+	+	+	+	+	+	—	7	No
McGann et al. [31]	+	+	+	+	+	—	+	—	6	Yes
Chan et al. [37]	+	+	+	+	+	+	+	+	8	Yes
Lee et al. [38]	+	+	+	+	+	+	+	+	8	Yes
Cohen et al. [39]	+	+	+	+	+	+	+	—	7	Yes
Laukkanen et al. [40]	+	+	+	+	+	—	+	+	7	No
Bonvin et al. [41]	+	+	+	+	+	+	+	+	8	No
Vernadakis [32]	+	+	+	+	+	—	+	—	6	Yes
Costello and Warne [42]	+	+	+	+	+	—	+	—	6	Yes
Johnson et al. [33]	+	+	+	+	+	—	+	—	6	No
Foweather et al. [43]	+	+	+	+	+	+	+	+	7	Yes
Sit et al. [44]	+	+	+	+	+	+	+	+	8	Yes
Lander et al. [45]	+	+	+	+	+	—	+	+	7	Yes
Lander et al. [46]	+	+	+	+	+	—	+	—	6	Yes
Johnstone et al. [47]	+	+	+	+	+	—	+	—	6	Yes
Karabourniotis et al. [48]	+	+	+	+	+	—	+	—	6	Yes
Mombarg et al. [34]	+	+	+	+	+	—	+	—	6	Yes
Aadland et al. [49]	+	+	+	+	+	—	+	+	7	Yes
Cliff et al. [50]	+	+	+	+	+	—	+	—	6	Yes
Pan et al. [51]	+	+	+	+	+	—	+	—	6	Yes
Piazza et al. [52]	+	+	+	+	+	—	+	—	6	Yes
Salmon et al. [53]	+	+	+	+	+	—	+	+	7	Yes
Sheehan and Katz [36]	+	+	+	+	+	—	+	—	6	Yes
Sheehan and Katz [35]	+	+	+	+	+	—	+	—	6	Yes
van der Fels et al. [54]	+	+	+	+	+	+	+	+	8	No

“+” refers to positive (explicitly described and present in details); “—” refers to negative (inadequately described and absent); “Yes” indicates significant positive effect; “No” indicates no significant effect. Median score = 7.
